# Comparative research on the development of college students’ physical fitness based on online physical education during the COVID-19 pandemic

**DOI:** 10.1186/s12889-023-15599-7

**Published:** 2023-04-21

**Authors:** Jun Sun, Jie Chang, Ergang Zhu, Xugui Sun, Yu Tao, Xiaohong Chen

**Affiliations:** 1 Department of Public Foundation, Wannan Medical College, Wuhu, China; 2 Department of Medical Information, Wannan Medical College, Wuhu, China; 3 College of Physical Education, Anhui Normal University, Wuhu, China

**Keywords:** Students, Online education, Physical education, Physical fitness

## Abstract

**Background:**

There are few studies that focus on the impact of online physical education teaching on college students during the coronavirus disease 2019 (COVID-19) pandemic. This research focuses on the impact of online physical education among medical school students in China by comparing physical fitness test results for three consecutive years from 2019 to 2021.

**Method:**

This study is a longitudinal survey. The subjects of the experiments were students enrolled in a medical school who completed a physical fitness test for three consecutive years from 2019 to 2021. The student subjects were divided into two groups, namely, male and female. The test indices included body mass index (BMI), vital capacity (VC), 50-metre run, sit-and-reach, standing long jump, pull-up (male), 1000-metre run (male), sit-ups (female) and 800-metre run (female). Repeated measures ANOVA method was used in physical fitness test indices at three consecutive time points ranging from 2019 to 2021. The Greenhouse-Geisser correction was applied when Mauchly’s hypothesis test did not meet the assumption of sphericity, and the Bonferroni method was used for pairwise comparisons.

**Results:**

A total of 3360 students (1490 males and 1870 females) completed physical fitness tests in three consecutive years from 2019 to 2021. The proportion of overweight and obesity in male students was significantly higher than that in female students (28.0% vs. 12.7%). For all subjects, in 2020, the BMI and VC indexes improved, while the 800-/1000-metre running indexes declined. In 2021, all indexes except sit-and-reach increased.

**Conclusion:**

The pairwise comparisons of physical fitness test results from 2019 to 2021 show that online physical education is effective in improving all items except long-distance running. Future research needs to involve a larger and geographically more dispersed sample to further analyse the effectiveness of online physical education.

## Background

The outbreak of coronavirus disease 2019 (COVID-19) at the end of 2019 disrupted conventional teaching worldwide [1]. In 2020, to meet government requirements that students and teachers should be isolated at home, colleges and universities suspended in-person teaching and adopted various forms of online teaching [2]. Although isolation effectively controlled the spread of COVID-19, it decreased daily physical activity and increased sedentary behaviour, which resulted in a decline in physical health [3, 4]. Studies [5, 6] have suggested that moderate physical activity is beneficial for alleviating the symptoms of COVID-19, reducing sedentary time, and improving physical health.

Online physical education (PE) has two advantages. First, there are rich teaching resources, including videos, slides and documents, available to support online PE. Second, online teaching transcends the limitations of region, time and space and makes physical education more visual, as students can watch videos repeatedly. However, regarding physical education teaching, orally communicating or watching videos is not enough; teachers need to guide students through body movements individually, which is difficult to do online [7]. Moreover, it is difficult for teachers to supervise students during online instruction [8].

To the best of our knowledge, there are few studies that focus on the impact of online physical education teaching on college students during the COVID-19 pandemic.The aim of our research is to provide insight into the impact of online physical education on the physical development of college students by comparatively analysing the physical fitness test results of students enrolled at Wannan Medical College for three consecutive years from 2019 to 2021, corresponding to the early (2019), middle (2020) and late (2021) periods of the pandemic; another aim is to further provide directions and suggestions for the future of physical education teaching.

## Method

The subjects of the experiment were 3360 college students at Wannan medical college (i.e., 1490 male students and 1870 female students) enrolled in 2019, with an age range from 17 to 22. Most of the students were from Anhui Province. The experiments were approved by the Research Ethics Review Committee of Wannan Medical College (Ref No:2022-093), and the students consent was obtained before the experiments. Physical fitness tests of all subjects were conducted from 2019 to 2021 and included indexes such as body mass index (BMI), vital capacity (VC), 50-metre run, sit and reach, standing long jump, pull-ups (male), 1000-metre run (male), sit-ups (female) and 800-metre run (female).

### The status quo of physical education at Wannan Medical College

In 2019, in-person physical education classes were held once a week for one and a half hours each time. The duration of each class included a 15-minute warm-up, 1-hour exercise and 15-minute cool-down. The outbreak of the pandemic at the end of 2019 prompted the launch of online physical education classes in 2020. During the two semesters that occurred in 2020,1-hour online physical education classes were carried out once a week with multiple options, including basketball, volleyball, football, tennis, handball, badminton, table tennis, aerobics, yoga, taekwondo and Chinese martial arts. In 2021, in-person physical education resumed.

### Measurement of physical fitness data

In China, students’ physical fitness is measured by an annual physical fitness test. In our experiments, to ensure fairness, the physical fitness tests were conducted by the same group of physical education teachers from Wannan Medical College. All tests were conducted from 8:00 am to 12:00 am and from 2:00 pm to 5:00 pm in December from 2019 to 2021. The following are items included in the annual physical fitness test.

#### BMI

BMI is calculated as the ratio of the weight in kilometres to the square of the height in metres. Height is measured by taking the length of students from the highest point of their head to their heel when they stand up straight after taking off their shoes. Weight is measured when students are wearing their usual clothing. According to the standards set by the World Health Organization (WHO), students are generally divided into four groups by BMI values, e.g., underweight (BMI<18.5 kg/m$^2$), normal (18.5<BMI<23.9 kg/m$^2$), overweight (24$\le$BMI<27.9 kg/m$^2$), and obese (BMI$\ge$28 kg/m$^2$).

#### VC

VC, as an important cardiorespiratory function estimator, uses a specialized spirometry tester to record the maximum volume of exhaled air after maximum volume of inhalation air.

#### 50-metre run

This index evaluates the speed and acceleration of students in seconds in a single 50-metre sprint.

#### Sit-and-reach

This index aims to assess flexibility. During the test process, students remain in a seated position with legs straight and hands stretched forwards as far as possible. By how much students’ hands exceed their feet is recorded in centimetres as the index value.

#### Standing long jump

The aim of this index is to assess explosive power by recording the length in centimetres when students try to jump as far as possible from a starting line.

#### Sit-up (female)

This index evaluates muscular endurance by recording the number of sit-ups completed within 60 seconds. During each sit-up, a female student holds her head, bends her legs from a supine position, and sits up as high as possible.

#### Pull-up (male)

To assess muscular endurance, this index records the number of pull-ups completed in 60 seconds. During each pull-up, a male student suspends from the horizontal bar with his hands, pulls himself up until his chin is above the horizontal bar, and then comes back down with his arms as straight as possible.

#### 800-metre run (female)/1000-metre run (male)

To assess muscular endurance, this index records the time in seconds it takes students to run 800/1000 metres on a standard track.

## Data analysis

Physical fitness test data was analysed by SPSS 27.0 (SPSS Inc.). The mean and standard deviation were used to quantify the quantitative variables, while the frequency and percentage were used to describe qualitative variables. Repeated measures ANOVA method was used in physical fitness test indices at three consecutive time points from 2019 to 2021. The Greenhouse-Geisser correction was applied when Mauchly’s hypothesis test did not meet the assumption of sphericity, and the Bonferroni method was used for pairwise comparisons.

## Results

A total of 3360 students (1490 males and 1870 females) completed physical fitness tests from 2019 to 2021.The statistics of the subjects are grouped by the test years (2019, 2020 and 2021) and shown in Table 1, which shows that for the three-year average, the overweight and obesity of male students was significantly higher than that of female students (28.0% vs. 12.7%), the normal weight of male students was lower than that of female students (60.9% vs. 72.9%), and the underweight of female students was higher than that of male students (23.0% vs. 11.1%).

**Table 1:** The basic characteristics of student subjects grouped by gender

Variables	Total			Male			Female		
	2019	2020	2021	2019	2020	2021	2019	2020	2021
		($n=3360$)			($n=1490$)			($n=1870$)	
Age (years)[n(%)]									
$\le$17	300(8.93)	25(0.74)	4(0.12)	115(7.71))	8(0.54)	0(0.00)	185(9.89)	17(0.91)	4(0.21)
18	1676(49.89)	275(8.18)	21(0.63)	727(48.79)	107(7.18)	8(0.54)	949(50.75)	168(8.98)	13(0.70)
19	1054(31.37)	1676(49.89)	275(8.18)	494(33.15)	727(48.79)	107(7.18)	560(29.95)	949(50.75)	168(8.98)
20	266(7.92)	1054(31.37)	1676(49.89)	124(8.32)	494(33.15)	727(48.79)	142(7.59)	560(29.95)	949(50.75)
21	52(1.55)	266(7.92)	1054(31.37)	26(1.74)	124(8.32)	494(33.15)	26(1.39)	142(7.59)	560(29.95)
$\ge$22	12(0.36)	64(1.90)	330(9.82)	4(0.27)	30(2.01)	154(10.34)	8(0.43)	34(1.82)	176(9.41)
Weight status [n(%)]									
Underweight	561(16.70)	607(18.07)	524(15.60)	194(13.02)	171(11.48)	132(8.86)	367(19.63)	436(23.32)	392(20.96)
Normal	2102(62.56)	2139(63.66)	2248(66.90)	851(57.11)	901(60.47)	968(64.97)	1251(83.96)	1238(66.20)	1280(68.45)
Overweight	496(14.76)	432(12.86)	428(12.74)	315(21.14)	291(19.53)	282(18.93)	181(12.15)	141(7.54)	146(7.81)
Obesity	201(5.98)	182(5.42)	160(4.76)	130(8.72)	127(8.52)	108(7.25)	71(4.77)	55(2.94)	52(2.78)
Post-hoc	P1<0.001;P2=0.001;P3=0.526			P1=1.000,P2=1.000,P3=1.000			P1<0.001,P2<0.001,P3=0.065		

*P*1, *P*2 and *P*3 mean the *P*-values of Post-hoc between 2019-2020, 2019-2021 and 2020-2021

The results show that for males, from 2019 to 2021, the proportions of underweight, overweight and obese students decreased, while the proportion of normal-weight students increased. For females, the proportions of normal-weight, overweight and obese students decreased during 2020 and then remained stable in 2021. The proportion of underweight individuals increased in 2020 and then rebounded in 2021.

Tables 2 and 3 compare the results of the physical fitness tests of male and female students over three consecutive years from 2019 to 2021. The two tables show remarkable similarities in male and female students. First, there were significant differences (*P *< 0.05) in the scores of male and female students in various tests in 2019 compared to 2020 and 2021. Second, compared with 2021, male and female students had significant differences in all test scores except those for sit-ups (*P *= 1.000) in 2020.

**Table 2:** The multiple comparative analysis of the three-year physical fitness tests on male students

Items				Post Hoc Multiple Comparison		
	2019(1)	2020(2)	2021(3)	1-2	1-3	2-3
Vital Capacity	4189(790)	4471(762)	4537(716)	<0.001	<0.001	<0.001
50-m sprint	7.5(0.5)	7.5(0.5)	7.4(0.5)	<0.001	<0.001	<0.001
Sit-and-reach	15.7(6.3)	16.6(6.5)	16.7(6.2)	<0.001	<0.001	1.000
Standing long jump	227(20)	229(20)	233(20)	<0.001	<0.001	<0.001
1000-m run	243(25)	249(26)	245(28)	0.001	<0.001	<0.001

**Table 3:** The multiple comparative analysis of the three-year physical fitness tests on female students

Items				Post Hoc Multiple Comparison		
	2019(1)	2020(2)	2021(3)	1-2	1-3	2-3
Vital Capacity	2733(519)	3025(517)	3087(509)	<0.001	<0.001	<0.001
50-m sprint	9.3(0.7)	9.2(0.6)	9.1(0.6)	<0.001	<0.001	<0.001
Sit-and-reach	18.9(5.5)	20.6(5.6)	20.6(5.3)	<0.001	<0.001	1.000
Standing long jump	172(16)	175(15)	178(16)	<0.001	<0.001	<0.001
800-m run	229(22)	236(22)	232(23)	<0.001	<0.001	<0.001
Bent-leg sit-up	33(8)	35(7)	37(7)	<0.001	<0.001	<0.001

**Fig. 1:**
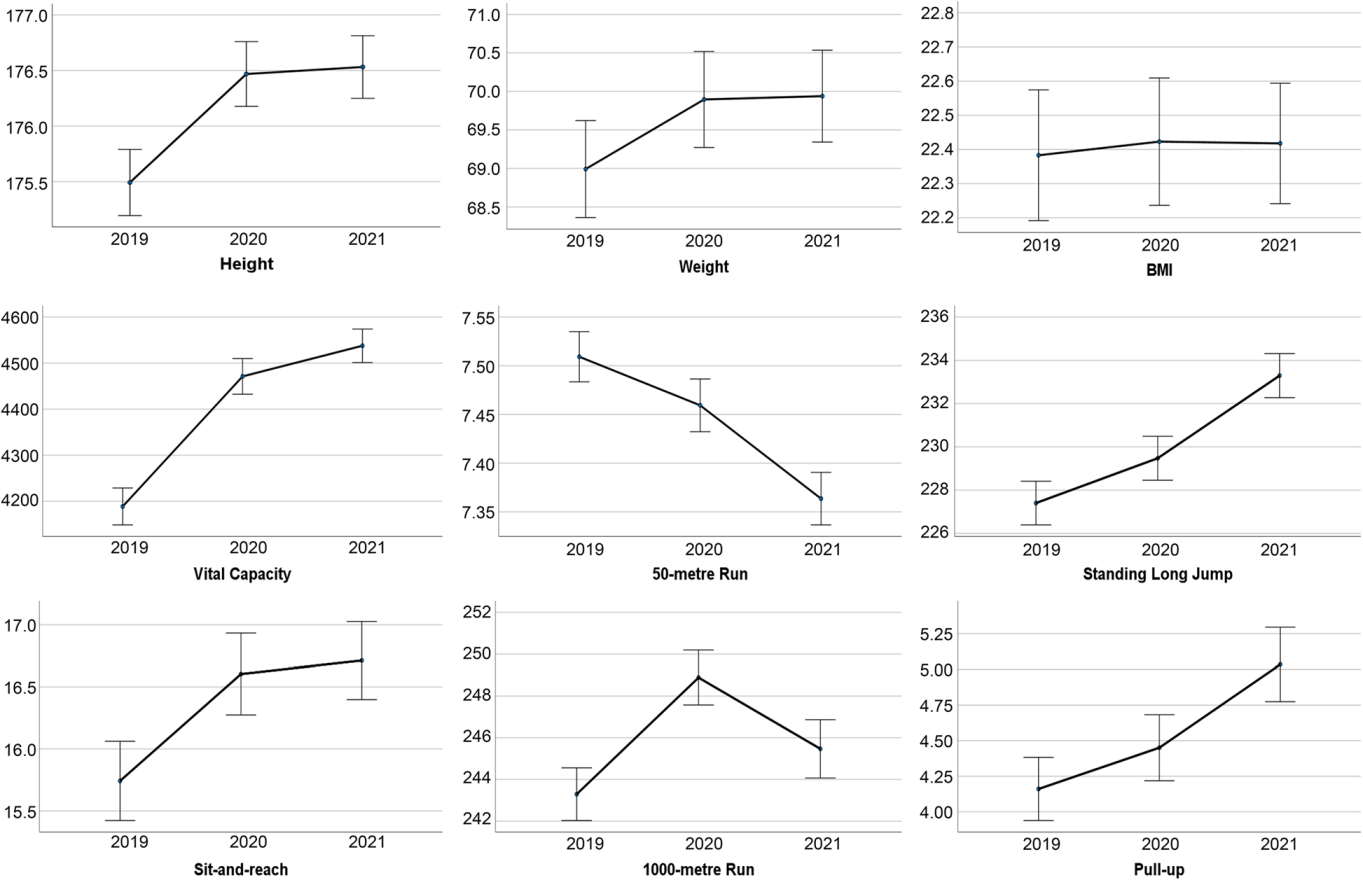
The average and 95% CI of items of the physical fitness tests on male students from 2019 to 2021

**Fig. 2:**
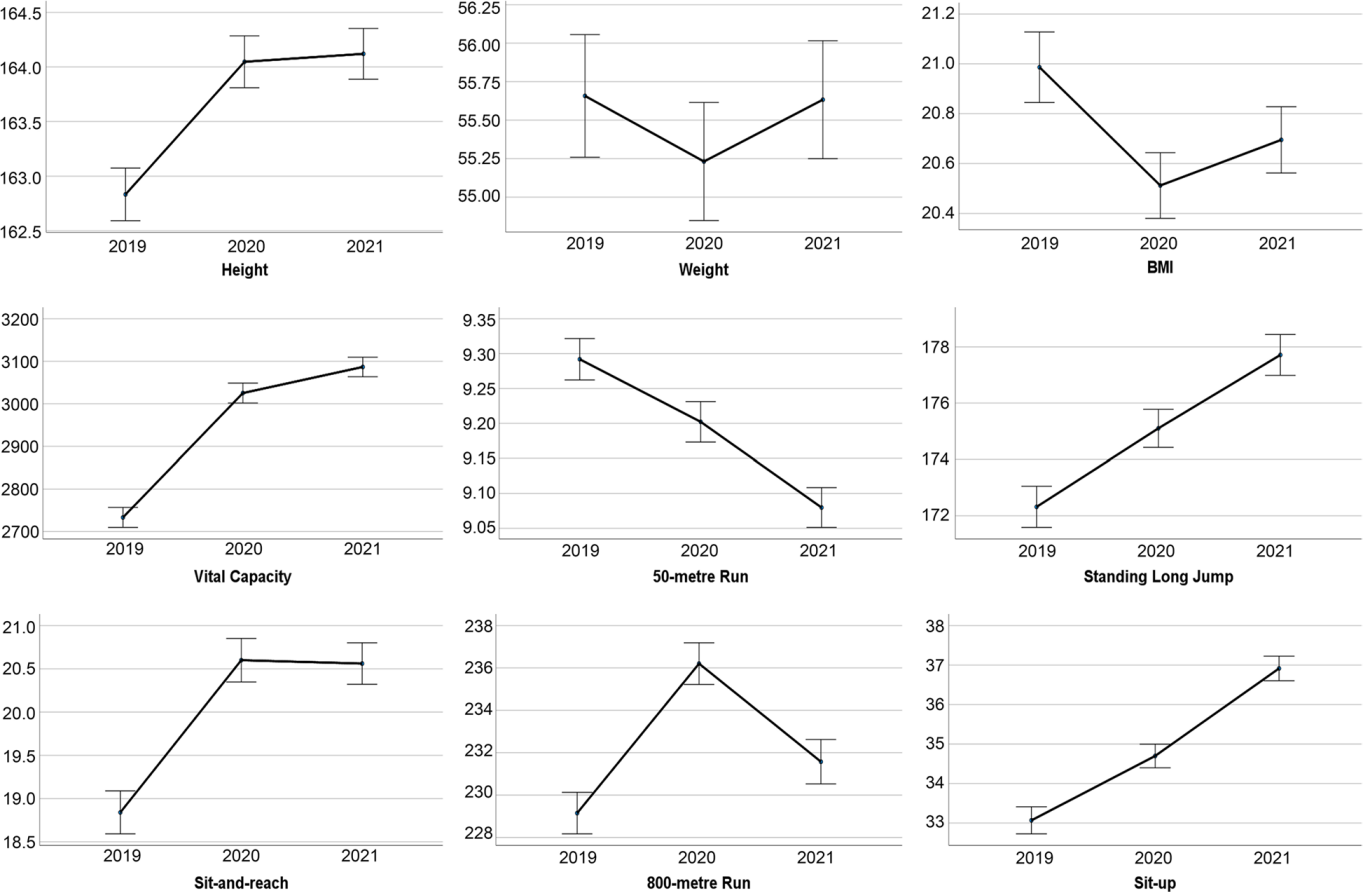
The average and 95% CI of items of the physical fitness tests on female students from 2019 to 2021

Figures 1 and 2 show the average and 95% CI of items of the physical fitness tests on male and female students from 2019 to 2021. Compared with 2020, in 2019, for male students, the lung capacity increased by 282 ml, the sit-and-reach increased by 0.9 cm, and the 1000-metre run decreased by 6 seconds. For female students, the vital capacity increased by 292 milliliters, the sit-and-reach increased by 1.7 cm, and the 800-metre run dropped by 7 seconds. Compared with 2021, in 2020, for male students, the mean vital capacity increased by 66 ml, the mean standing long jump increased by 4 cm, and the mean time of 1000-metre run increased by 4 seconds. For female students, the mean time of 50-metre run increased by 0.1 seconds, and the mean time of 800-metre run increased by 4 seconds.

## Discussion

The purpose of this study is to provide deep insight into the impact of online physical education on the physical fitness of college students during the pandemic. Physical fitness tests were conducted on students enrolled at a university in Anhui Province in China during December of 2019, 2020 and 2021. We found that the number of overweight and obese female students has declined since the COVID-19 outbreak in 2019, which is worthy of attention. The resulting analysis also shows that for all student subjects, lung capacity has increased significantly, while the performances in the 800/1000-metre runs have decreased sharply; these results are consistent with the results of the XIA study [9]. This study fills a gap in the research on the impact of the pandemic on physical education. The results show that online physical education teaching is effective in improving the physical fitness of college students.

Since college students are still growing adolescents, male and female subjects showed development in terms of body composition indices (height and weight). First, existing studies have shown that the pandemic has led to a reduction in students physical activities [10], an increase in their sedentary time, and changes in their eating habits, all of which affect the BMI of students [11, 12]. Our study shows that for male students, the trend of BMI being normal increased from 2020 to 2021. This phenomenon indicates that in-person physical education is more effective than online sports education in normalizing the BMI index. Compared with male students, the number of overweight and obese female students remained stable in 2021. Overall, the BMI of male students continued to improve from 2020 to 2021, which is consistent with the results reported by Hallal [13] that males are more active in sports than females.

Studies have found that the lung capacity of all subjects improved significantly in 2020. However, some studies suggest that during the pandemic, less exercise may have led to lower lung capacity, making people susceptible to cardiometabolic diseases and other adverse outcomes [14]. This may indicate that online physical education helps college students either maintain or increase their physical activity level, thereby increasing their lung capacity. In 2021, lung capacity continued to improve, but at a lower rate than in 2020.

In our study, except for the 800-/1000-m run, there was a significant improvement in the scores of the 50-m run, sit-ups, standing long jump, pull-ups (males), and sit-and-reach (females) in 2020. The reason is that under the condition of isolation, these exercises can be conducted conveniently, which is consistent with the results indicated in Fearnbach [15]. This outcome may also indicate that online physical education is effective in improving the performance of these items. In 2021, when in-person physical education resumed, all items except for the sit-and-reach improved significantly, which not only indicates that online physical education is effective in improving students’ flexibility, but also suggests a lack of flexibility training in offline physical education.

We also found that the indices of the 1000-metre run (male) and 800-metre run (female) tests dropped significantly in 2020 and then increased sharply when in-person PE resumed in 2021. Especially for females, the 800-metre index returned to the level seen in 2019. This result implies that online physical education is unbeneficial in improving students’ muscular endurance quality. The main reasons for this result may be as follows. First, the limitations of the venues available during the pandemic made conventional endurance training methods such as long-distance aerobic running and slow running difficult to carry out. Second, students have little knowledge of home-based endurance training methods, and online physical education has not yet focused on these methods. Finally, for most students, the habit of self-exercise at home has not been developed .

Overall, the replacement of in-person physical education with its online counterpart in 2020 had negative effects on pull-ups and 800-/1000-metre runs but had significantly positive effects on other items; these findings are consistent with the XIA research report [9]. The reason is that the three aforementioned items are strongly related to the outdoor training environment. We suggest that online physical education should choose more convenient and practical home exercises to improve students’ muscular endurance, such as TikTok dancing, sprinting in place and shuttle running [16].

This study summarizes the practical experience of online physical education teaching adopted by a university during the epidemic, which is important for improving the theoretical system of higher education. At the same time, online teaching is of great practical significance to completing high-quality teaching tasks during public health incidents [7].

This study has two main strengths. First, the study had a sample size of 3360 student subjects (1490 males and 1870 females), which was larger than most previous studies in the field and allowed for greater statistical power and more reliable results. Second, the study reviewed physical fitness tests of the same students over a period of three years, which provided a more comprehensive understanding of the phenomenon under study. The longitudinal design allowed for the identification of trends and patterns in the data that may not have been apparent with a cross-sectional design. However, we admit that this study still has limitations. First, the subjects in this study were not sufficient in regard to quantity and regional dispersity. Second, the confounding factors of the subjects, such as their choice of physical education courses, the completion of their physical homework, their living standards and living styles, their mental state, etc., were not controlled in our experiments.

## Conclusion

This study focuses on a comparative analysis of the changes in physical fitness tests from 2019 to 2021 as a result of online physical education teaching intervention in 2020. Online physical education teaching played a significant role in improving the physical fitness of college students during the COVID-19 epidemic. However, this approach has limitations in regard to developing students’ muscular endurance due to site-specific constraints. In the future, the combination of online and offline teaching, which can make the most of both teaching modes, is worth studying in regard to PE instruction.

## Data Availability

The datasets used and/or analyzed during the current study available from the corresponding author on reasonable request.
